# Up-Regulation of CREG Expression by the Transcription Factor GATA1 Inhibits High Glucose- and High Palmitate-Induced Apoptosis in Human Umbilical Vein Endothelial Cells

**DOI:** 10.1371/journal.pone.0154861

**Published:** 2016-05-03

**Authors:** Yanxia Liu, Xiaoxiang Tian, Yang Li, Dan Liu, Meili Liu, Xiaolin Zhang, Quanyu Zhang, Chenghui Yan, Yaling Han

**Affiliations:** 1 Graduate School of Third Military Medical University, Chongqing, China; 2 Department of Cardiology and Cardiovascular Research Institute, General Hospital of Shenyang Military Region, Shenyang, China; Thomas Jefferson University, UNITED STATES

## Abstract

**Background:**

Endothelial cell (EC) apoptosis plays a vital role in the pathogenesis of atherosclerosis in patients with diabetes mellitus (DM), but the underlying mechanism remains unclear. Cellular repressor of E1A-stimulated genes (CREG) is a novel gene reported to be involved in maintaining the homeostasis of ECs. Therefore, in the present study, we investigated the role of CREG in high glucose/high palmitate-induced EC apoptosis and to decipher the upstream regulatory mechanism underlying the transcriptional regulation of CREG.

**Methods:**

The expression of CREG and the rate of apoptosis were assessed in lower-limb atherosclerotic lesions from patients with type 2 DM (T2DM). Primary human umbilical vein endothelial cells (HUVECs) were isolated and cultured in a high glucose/high palmitate medium (25 mmol/L D-glucose, 0.4 mmol/L palmitate), and the over-expression and knock-down of CREG were performed in HUVECs to determine the role of CREG in EC apoptosis. The upstream regulatory mechanism of CREG was identified using a promoter-binding transcription-factor profiling array, chromatin immunoprecipitation (ChIP) assay and a mutation analysis.

**Results:**

Compared with normal arteries from non-diabetic patients, reduced CREG expression and increased apoptosis were found in the endothelium of atherosclerotic lesions from patients with T2DM. In vitro treatment of HUVECs with a high glucose/high palmitate medium also resulted in decreased CREG expression and increased apoptosis. Moreover, high glucose/high palmitate induced-HUVEC apoptosis was increased by the knock-down of CREG and rescued by the over-expression of CREG. We also demonstrated that GATA1 was able to bind to the promoter of the human CREG gene. A deletion mutation at -297/-292 in the CREG promoter disrupted GATA1 binding and reduced the activation of CREG transcription by approximately 83.3%. Finally, the overexpression of GATA1 abrogated the high glucose/high palmitate-induced apoptosis in HUVECs.

**Conclusions:**

The over-expression of CREG inhibits high glucose/high palmitate-induced apoptosis in HUVECs. CREG is transcriptionally upregulated by GATA1. Thus, CREG might be a potential therapeutic target for intervention of vascular complications related to diabetes.

## Background

Macrovascular complications [e.g., coronary artery disease, peripheral vascular disease (PVD) and stroke] are the major causes of mortality in patients with type 2 diabetes mellitus (T2DM), accounting for more than 50% of diabetes-related deaths[1]. Diabetic macrovascular complications usually feature accelerated atherosclerosis, which is anatomically similar to atherosclerosis in nondiabetic individuals but is more extensive and occurs much earlier[2, 3]. A growing body of evidence suggests that endothelial dysfunction and apoptosis are the earliest and the most critical events in the onset and progression of atherosclerosis in diabetic patients[4, 5]. During the progression of DM, endothelial cells (ECs) exposed to hyperglycaemia and hyperlipidaemia undergo apoptotic processes, leading to the detachment of endothelial cells from the intima. This initial denudation consequently triggers pro-atherosclerotic responses, resulting in the development of atherosclerosis and macrovascular complications in those with DM[6, 7]. Although a causal relationship between endothelial apoptosis and atherosclerosis is well established, the underlying mechanisms remain incompletely understood. Therefore, there is an increasing demand for investigations into new therapeutic targets directed at reducing EC apoptosis and atherosclerosis in diabetic patients.

Cellular repressor of E1A-stimulated genes (CREG) is a novel glycoprotein that is highly expressed in blood vessels under physiological conditions[8]. It has been well established that CREG plays essential roles in maintaining homeostasis in vascular smooth muscle cells by regulating their proliferation, migration and phenotypic switches[9–12]. Notably, results from our lab have also demonstrated that CREG is critically involved in the differentiation and functional maintenance of ECs[13, 14]. CREG has been found to be abundant in both the arterial endothelium and in primary human umbilical vein endothelial cells (HUVECs)[15], implying its potential importance. We have also confirmed that CREG promotes the proliferation and migration of HUVECs through the activation of different signalling pathways[16, 17]. Moreover, CREG is essential for in vivo and in vitro angiogenesis[18, 19]. In a porcine coronary injury model, the implantation of a CREG-protein-eluting stent resulted in the acceleration of the re-endothelialization of the coronary artery[20]. In a tumour necrosis factor alpha-induced EC inflammation model, the over-expression of CREG inhibited the inflammatory responses and the hyper-permeability of ECs[14]. In addition, CREG inhibits endothelial apoptosis via the VEGF/PI3K/Akt pathway[15]. Finally, CREG has also been reported to be involved in the apoptosis of vascular smooth muscle cells and bone marrow mesenchymal stem cells[10, 21]. Considering the above role of CREG in ECs and in apoptosis, we aimed to determine whether CREG aetiologically participates in EC apoptosis under conditions associated with DM.

In the present study, we chose an in vitro model of genetically modified HUVECs and exposed them to high glucose/high palmitate concentrations to explore the specific role of CREG in diabetes-associated EC apoptosis. To elucidate the upstream transcriptional regulation of CREG expression, we generated luciferase constructs to determine the core promoter of the CREG gene. Then a promoter-binding transcription-factor profiling array assay, a chromatin immunoprecipitation (ChIP) assay and mutations of the binding site were performed to determine the upstream transcriptional factors involved. Finally, the role of key transcriptional factor GATA1 in regulating the expression of CREG and high glucose/high palmitate-induced ECs apoptosis were determined. Our results demonstrate that the over-expression of CREG inhibits in HUVECs and that CREG is transcriptionally upregulated by GATA1. Thus CREG might be a potential therapeutic target for management of vascular complications in patients with DM.

## Methods

### Ethics approval and consent to participate

The use of human blood vessels and umbilical cords was approved by the Ethics Committee of General Hospital of Shenyang Military Region, and written informed consent was obtained from all patients. All animal experiments conformed to the Principles of Laboratory Animal Care formulated by the National Society for Medical Research and the Guide for the Care and Use of Laboratory Animals [NIH Publication 86–23, 1985 revision] and were approved by the Ethics Committee on the Care and Use of Laboratory Animals of General Hospital of Shenyang Military Region.

### Materials

Human Endothelial Culture Medium was purchased from Thermo Scientific (Grand Island, NY, USA). The FITC-Annexin V/PI Apoptosis Detection Kit was obtained from BD Biosciences (Franklin Lakes, NJ, USA). The Promoter-Binding TF Profiling Plate Array I (FA-2001) was obtained from Signosis (Santa Clara, CA, USA). Dual-Luciferase Reporter Assay System (E1960), PGL4.12 (E6671) and PGL4.73 (E6911) vectors were purchased from Promega (Madison, WI, USA). The ChIP assay kit was obtained from Millipore (Billerica, MA, USA). Plasmid pcDNA3.1 (+), a terminal deoxynucleotidyl transferase-mediated dUTP-biotin nick end-labelling (TUNEL) kit and TRIzol Reagent were purchased from Invitrogen (Grand Island, NY, USA). Antibodies against CREG (ab191909, ab68341) and GATA1 (ab181544) were purchased from Abcam (Cambridge, MA, USA). Antibodies against cleaved caspase-3 and GAPDH were obtained from Cell Signalling Technology (Danvers, MA, USA). The enhanced chemiluminescence (ECL) Western Blotting System was purchased from GE Life Sciences (Marlborough, MA, USA). D-glucose, free fatty acid (FFA)-free bovine serum albumin (BSA), palmitate and other chemicals, if not specified otherwise, were purchased from Sigma-Aldrich (St Louis, MO, USA).

### Patients and blood vessels

Lower extremity arterial blood vessels were obtained from amputees with diabetic PVD (n = 4) or from individuals who had suffered a traffic accident without PVD and diabetes (n = 4) enrolled between April 2015 and December 2015. Subjects were considered eligible if: (1) they were age 18 years or older, (2) they were awaiting a transtibial or transfemoral amputation, and (3) the primary cause of amputation was complications of PVD and diabetes or motor vehicle crashes without PVD or diabetes. Subjects were excluded if: (1) they had inadequate cognitive or language function to consent or participate, and (2) there was significant history of extremity trauma or tumour. The use of human vessels was approved by the Ethics Committee of the General Hospital of Shenyang Military Region and informed consents were also obtained from all patients. A total of ten patients approached for donation, two of them were excluded and eight of them met the study criteria to be enrolled. The collected human diseased and control arteries were properly treated for further analysis of CREG expression and apoptosis via immunostaining and immunoblotting.

### Cell culture and apoptosis analysis

Primary HUVECs were isolated from umbilical cords using 0.2% collagenase II and grown in endothelial culture medium supplemented with 10% fetal bovine serum, 20 ng/ml basic fibroblast growth factor, 10 ng/ml epidermal growth factor, 10 μg/ml human plasma fibronectin, 100 U/ml penicillin, and 100 μg/ml streptomycin at 37°C in a 5% CO_2_ atmosphere as described previously[16]. HUVECs were passaged by 0.25% trypsin digestion when they reached confluence. The use of umbilical cords was approved by the Ethics Committee of the General Hospital of Shenyang Military Region and informed consents were obtained. For high glucose and high FFA conditions, HUVECs were incubated in medium supplemented with 25 mM D-glucose and 0.2~0.4 mM BSA-conjugated palmitate[22, 23]. HUVECs cultured in medium containing 5.5 mM D-glucose, 19.5 mM L-glucose and 0.4 mM BSA were used as normoglycemic and normal FFA controls. Cells were treated for 24 h prior to harvesting for Western blotting and mRNA analysis. Apoptosis was assessed via fluorescence activated cell sorting (FACS, BD FACS Calibur, USA) and TUNEL staining according to the manufacturer’s instructions provided with the FITC-Annexin V/PI Apoptosis Detection kit and the TUNEL staining kit as described previously[15]. HUVECs from passages 3–8 were used in all experiments.

### Genetic modification of HUVECs

HUVECs over-expressing CREG (CREG-OE) and cells with CREG expression knocked down (CREG-KD) were generated as described previously[15]. In brief, retroviruses expressing human CREG (hCREG) and short hairpin RNAs (shRNAs) targeting the open reading frame of hCREG were used to infect HUVECs. Infected cells were selected by 500 μg/ml G418 for 7 days to obtain CREG-OE or CREG-KD HUVECs. HUVECs infected with retroviruses expressing scrambled shRNA were used as a normal control (CREG-NR).

### Haematoxylin-eosin (HE) staining and immunofluorescence staining

For HE staining, arteries were washed with an ice-cold phosphate-buffered saline (PBS) solution, fixed with 4% buffered paraformaldehyde, embedded in paraffin, prepared as 2-μm-thick sections and analysed histologically via HE staining.

For the immunofluorescent staining of the arteries, samples were washed with ice-cold PBS solution, fixed with 4% buffered paraformaldehyde, dehydrated in 20% sucrose at 4°C overnight, embedded in OCT compound, snap-frozen in liquid nitrogen, and prepared as 5-μm-thick sections. Then, tissue sections were permeabilized with 0.2% Triton-X100. After washing the sections with PBS three times, nonspecific binding sites were blocked with 5% BSA for 30 min at room temperature. The samples were then incubated with mouse anti-CREG monoclonal antibodies (Abcam, ab68341, 1:100) at room temperature for 1 h, followed by incubation with Alexa Fluor 594 conjugated donkey anti-mouse secondary antibodies (Jackson Immuno Research, 1:100) for 30 min at room temperature in a humidified box. Nuclei were stained with 4’, 6-diamidino-2-phenylindole (DAPI). Stained sections were observed and images were obtained under a DM 3000 microscope (Leica Microsystems, Germany).

### Real-time PCR and Western blot analysis

For real-time PCR analysis, total RNA was isolated using TRIzol Reagent. Reverse transcription was performed at 50°C for 50 min, and then at 85°C for 5 min using the SuperScript III First-Strand Synthesis SuperMix for RT-PCR kit (Invitrogen, CA, USA). Real-time PCR was performed on an ABI 7300 Real-Time PCR system (Applied Biosystems, USA) as described previously[24]. The primers used for the amplification of hCREG were 5’-GGCGTGCCCTATTTCTACCTG-3’ (forward) and 5’-TTTCTTGCAGAAGTTGGTCTGT-3’ (reverse). The primers used for GAPDH amplification were 5’-CCAGGCGCCCAATACG-3’ (forward) and 5’-CCACATCGCTCAGACACCAT-3’ (reverse).

For the Western blot analysis, dissected human arteries were homogenized and cell aggregates were lysed in an RIPA buffer (Thermo Scientific, USA) containing protease and phosphatase. The cleared supernatant was collected, and the protein concentration was determined using the BCA Protein Assay Kit (Thermo Scientific, USA). Western blotting was performed as previously described[24]. The blotting films were captured, and bands were quantified using Quantity One software version 4.6 (Bio-Rad Laboratories, Hercules, CA, USA).

### Construction of the hCREG promoter-dual-luciferase reporter genes with various deletions and the promoter activity study

Various lengths of the hCREG gene’s 5’-flanking region containing 2003 bp (−1925/+78), 945 bp (−867/+78), 586 bp (−508/+78), 478 bp (−400/+78) and 358 bp (−280/+78) with the XohI/HindIII restriction sites were either synthesized or amplified via PCR (Takara, Dalian, China). The transcription start site (TSS) is designated as “+1” through-out the text and translation start site (ATG) is located at 78 bp from TSS (+78). All of the fragments were confirmed via DNA sequencing. Then, fragments of various length of the hCREG 5’-flanking region were inserted into promoterless dual luciferase reporter plasmids (pGL4.12-basic; Promega, Madison, WI, USA). The promoter activity of the hCREG constructs was examined in cultured HUVECs and 293T cells using a transient transfection and a luciferase assay. Plasmids (pGL4.73) expressing renilla luciferase were co-transfected to correct for variations in transfection efficiency. The ratio of firefly luciferase activity to renilla luciferase activity in each transfection was used as the normalized luciferase activity to quantify promoter activity. To confirm the binding sites of GATA1, the two consensus GATA1 binding sequences (TATCTC and CAGATA) in the promoter region of hCREG (−508/+78) were deleted via PCR to generate mutant fragment 1 (GATA1-MUT1) and mutant fragment 2 (GATA1-MUT2). The two mutant fragments were then inserted into the reporter vectors, and promoter activity was measured as described above. Each construct was transfected three times, and each transfection was performed in triplicate. Data for each construct are presented as the mean ± SE.

### Promoter-Binding Transcription-Factor Profiling Array assay

To screen for transcription factors (TFs) that bind to the hCREG promoter (-508/+78), the activities of 48 TFs in HUVECs were assayed using a Promoter-Binding TF Profiling Array (Signosis, CA). This is a competitive binding assay in which nuclear extract is incubated with 48 different biotin-labelled oligonucleotide probes. Each oligo probe can bind to a single TF. After incubation, TF-bound complexes are eluted, denatured and hybridized with the complementary DNA of the specific probe to be captured. After capture, oligos are detected using streptavidin-HRP and a chemiluminescent substrate. If unlabelled promoter DNA fragments of interest contain a TF binding sequence, they will compete with the biotin-labelled oligo to bind to the TF in the sample, leading to no or decreased biotin-labelled TF/DNA complex formation and to no or decreased detection. Based on a comparison of the results in the presence and absence of the competing promoter DNA fragment of interest, promoter-bound TFs can be identified.

Assay was performed as recommended by the manufacturer’s instructions. Briefly, nuclear extract was isolated from 10^6^ HUVECs using a nuclear protein extraction kit (Thermo). The reaction mixture was prepared using 15 μl of the TF binding buffer, 3 μl of the probe, 10 μg of nuclear extract and 5 μl of the hCREG promoter fragment (-508/+78) and was incubated at room temperature for 30 min to allow for the formation of the TF-DNA complex. Unbound probes were separated from the complex, while bound probes were eluted and then hybridized to the plate and incubated overnight at 42°C. Bound probe was detected using an HRP-streptavidin conjugate incubated with the chemiluminescent substrate. Luminescence is reported as relative light units (RLUs) on a microplate luminometer (Wallac 1450, Wallac, MA, USA).

### ChIP assay

ChIP assays were performed using a ChIP assay kit as per the manufacture’s instruction. The two sets of PCR primers used for the predicted GATA1 binding sequence in the hCREG promoter region (-508/+78) were as follows: ChIP1 (-599~-412 bp), 5’- CCTCGATTCCCTGAAACTCCC -3’ (forward) and 5’- CAGCTACACATGGGGTACACG -3’ (reverse) and ChIP2 (-432~-267 bp), 5’-CAGCTACACATGGGGTACACG-3’ (forward) and 5’-TTCATCTCCAAAGGTATTAAAAGGA-3’ (reverse).

### Statistical Analysis

Data are expressed as the mean ± SE. Statistical analyses were performed using SPSS version 19.0 (SPSS, Inc., Chicago, Illinois). Student’s t test was applied for comparing two groups, and a one-way ANOVA with Tukey’s post hoc analysis was used for comparing more than two groups. A p value of < 0.05 was considered to be statistically significant.

## Results

### Expression of CREG is reduced at atherosclerotic-prone sites associated with increased endothelial apoptosis in human diabetic arteries

**Fig 1:**
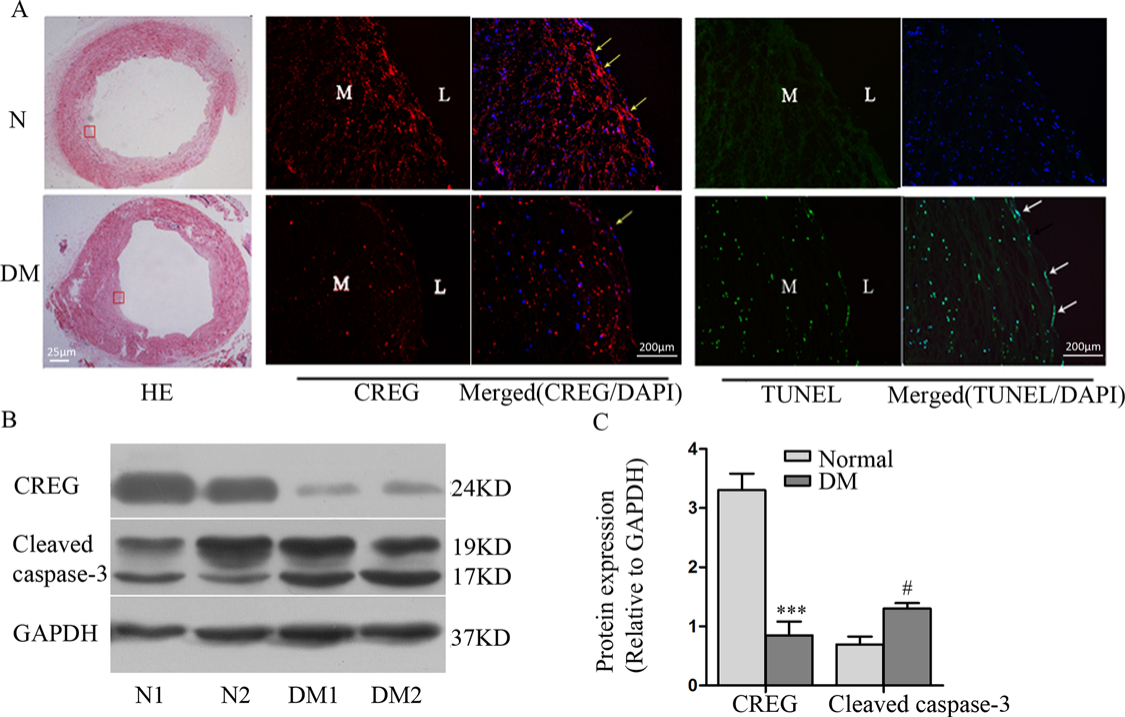
Expression of CREG was shown to be dramatically reduced in arteries from diabetic patients and to be associated with increased endothelial apoptosis. (A) HE staining (left panel) shows that arteries from patients with diabetic mellitus (DM) presented with significant atherosclerotic plaques, while arteries from patients without diabetes (N) had a relatively normal intima. Immunofluorescence staining of CREG (middle panel) and TUNEL staining (right panel) show that CREG expression was reduced, while the number of apoptotic (TUNEL-positive) cells was increased in DM arteries relative to the results in the control arteries. Yellow arrows indicate CREG, and white arrows indicate apoptotic endothelial cells. Blue: cell nuclei (DAPI staining); L: lumen; M: media. (B) Expression of CREG and cleaved caspase-3 in non-diabetes (N1 and N2) and atherosclerotic arteries of diabetic patients (DM1 and DM2) were detected via Western blotting. Blots were quantified via densitometry and plotted as a ratio relative to GAPDH. n = 4. ***P<0.001 and #P<0.05 compared with the normal control.

To explore the relationship between CREG and endothelial apoptosis under diabetic conditions, we detected CREG expression and endothelial apoptosis in atherosclerotic arteries from diabetic amputees and in control arteries from DM-free amputees. HE staining showed that the arteries used in this study from diabetic patients presented with significant atherosclerotic plaques, while arteries from patients without diabetes showed a relatively normal intimal structure (Fig 1A). In addition, immunofluorescence staining showed that CREG expression was significantly reduced in the endothelium of diabetic arteries compared with the expression in control arteries. TUNEL staining further showed that apoptosis occurred more frequently in the endothelium of diabetic arteries than in the control arteries (Fig 1A). Immunoblotting also demonstrated significantly decreased CREG expression and increased apoptosis in diabetic arteries (Fig 1B). These data suggest that CREG might be involved in endothelial apoptosis under diabetic conditions.

### High glucose/high palmitate concentrations increases apoptosis in primary HUVECs

**Fig 2:**
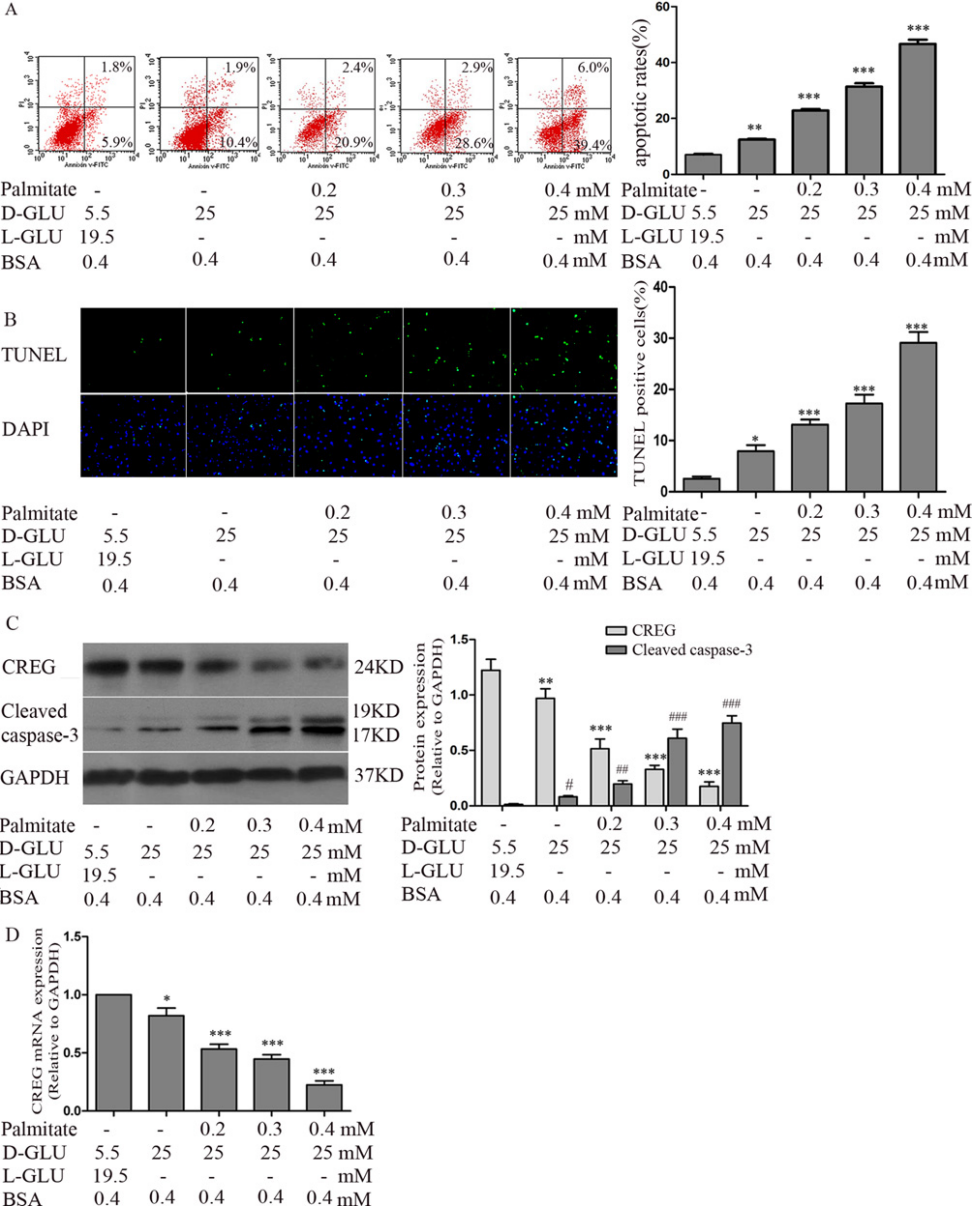
High glucose and high palmitate concentrations induced HUVEC apoptosis. HUVECs treated with high glucose (25 mM D-glucose) and a range of palmitate (0.2, 0.3 and 0.4 mM) concentrations were established to simulate pathological stimuli experience with diabetes. HUVECs incubated with 5.5 mM D-glucose,19.5 mM L-glucose and 0.4 mMBSA were used as normoglycaemic controls. Cells were treated for 24 h prior to the following analyses. (A) Apoptosis was assessed via Annexin V/PI dual-colour flow cytometry. Cells staining positive for Annexin V-FITC and negative for PI were considered to be undergoing apoptosis. (B) Apoptosis was assessed via TUNEL staining. (C) Expression of CREG and cleaved caspase-3 were determined via immunoblotting. (D) Expression of CREG and cleaved caspase-3 were determined via real-time PCR. GAPDH served as a control. n = 6. All experiments were performed in triplicate. Data are shown as the mean ± SE. *, #P<0.05, **, ##P<0.01 and ***, ###P<0.001 compared with the normoglycemic control.

To elucidate the role of CREG in endothelial apoptosis, an in vitro model of HUVECs treated with high glucose (25 mM D-glucose) and a range of palmitate concentrations (0.2, 0.3 and 0.4 mM) was established to mimic the pathological stimuli present in individuals with DM. Annexin V/PI dual-colour flow cytometry showed that compared with the normoglycemic control (5.5 mM D-glucose, 19.5 mM L-glucose and 0.4 mM BSA), high glucose alone increased HUVEC apoptosis (14.23±3.39% vs 6.35±2.82%, P<0.01), and the combination of high glucose with different concentrations of palmitate (0.2, 0.3 and 0.4 mM) further increased cell apoptosis in a dose-dependent manner (23.19±6.42%, 31.48±4.60% and 48.43±6.70%, respectively, P<0.001) (Fig 2A). Endothelial apoptosis was also confirmed by detecting DNA fragments via TUNEL staining. Similarly, high glucose with a range of palmitate concentrations (0.2, 0.3 and 0.4 mM) increased the percentage of TUNEL-positive cells in a dose-dependent manner (13.10±2.50%, 17.24±4.31% and 29.12±5.11%, respectively), and this combined effect was greater than that observed with high glucose alone (7.92±2.90%) and in the normoglycemic control (3.25±1.80%) (Fig 2B). Moreover, immunoblotting showed that the expression of CREG was reduced, while cleaved caspase-3 expression was gradually increased after treatment with high glucose and with high glucose plus palmitate at various concentrations (Fig 2C). In addition, RT-PCR analysis showed that the level of CREG mRNA was decreased in a pattern similar to that observed for the expression of the CREG protein (Fig 2D), indicating that the regulation of CREG expression occurred at the level of transcription. Because high glucose with high palmitate (0.4 mM) produced the most significant pro-apoptotic effect and CREG reduction, it was employed in the following experiments. The above in vitro data accurately reflect the changes in CREG expression and apoptosis observed in vivo from diabetic arteries, justifying further investigation into the causal effect of CREG in the process of endothelial apoptosis under diabetic conditions.

### High glucose/high palmitate-induced reduction of CREG expression caused apoptosis in HUVECs

**Fig 3:**
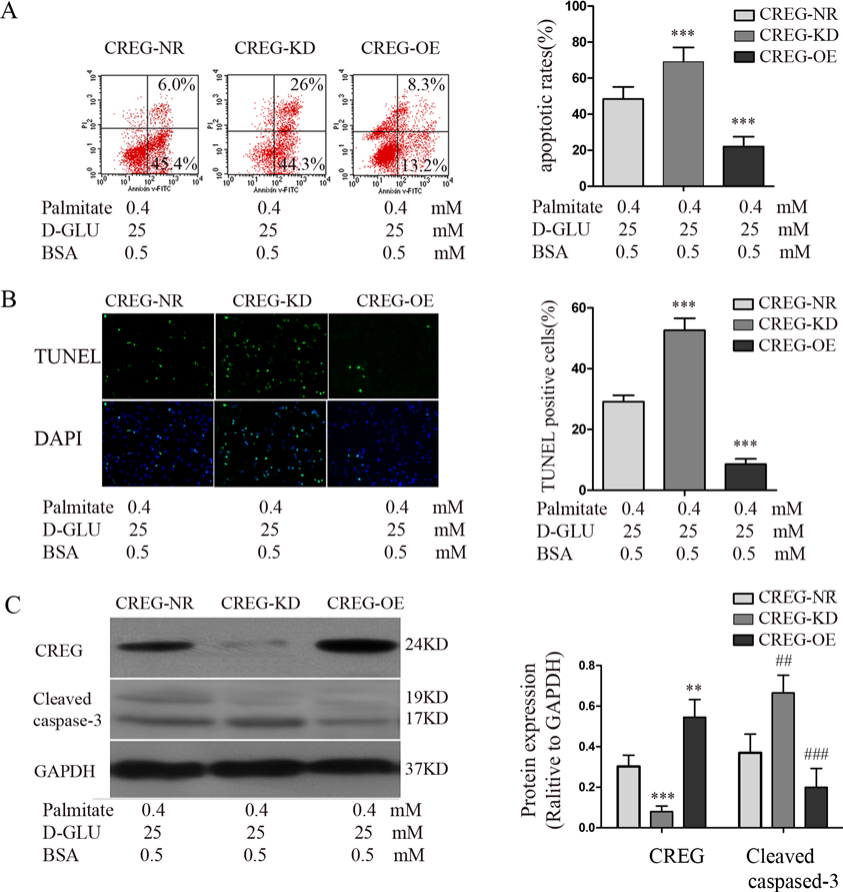
Effect of CREG expression level on high glucose/high palmitate-induced apoptosis in HUVECs. HUVECs over-expressing CREG (CREG-OE) and CREG knock-down cells (CREG-KD) were established, and HUVECs expressing scrambled shRNA sequences were used as a normal control (CREG-NR). Cells were treated with 25 mM D-glucose and 0.4 mM palmitate for 24 h prior to the following analyses. (A) Apoptosis was assessed via Annexin V/PI dual-colour flow cytometry. Cells staining positive for Annexin V-FITC and negative for PI were considered to be undergoing apoptosis. (B) Apoptosis was assessed via TUNEL staining. (C) Expression of CREG and cleaved caspase-3 were determined via immunoblotting. (D) Expression of CREG and cleaved caspase-3 were determined via real-time PCR. GAPDH served as a loading control. All experiments were performed in triplicate. n = 6. Data are shown as the mean ± SE. **, ##P<0.01 and ***, ###P<0.001 compared with the CREG-NR group.

To determine whether a causal relationship exists between CREG and EC apoptosis, we established CREG-OE, CREG-KD, and CREG-NR as previously described. Then, apoptosis and the expression of CREG in three groups of HUVECs treated with high glucose (25 mM D-glucose) and high palmitate (0.4 mM) for 24 h were assessed. Annexin V/PI flow cytometry showed that the percentage of apoptotic cells was dramatically increased (69.13±3.23%) in the CREG-KD cells and was significantly decreased in CREG-OE cells (22.03±2.27%) relative to the expression in CREG-NR HUVECs (48.43±2.73%) (Fig 3A). TUNEL staining also showed results similar to the cytometry findings in CREG-KD (52.60±3.95%), CREG-OE (8.55±1.76%) and CREG-NR (29.12±5.11%) cells, as shown in Fig 3B. Western blotting demonstrated that the expression of cleaved caspase-3 was remarkably decreased in CREG-OE cells and was increased in CREG-KD cells (Fig 3C). These data provide direct evidence that high glucose/high palmitate-induced endothelial apoptosis is caused by a reduction of CREG, which can be rescued by the over-expression of CREG. Thus, CREG might be a therapeutic target for altering endothelial apoptosis and atherosclerosis in patients with DM, and exploring factors which can directly regulate CREG expression is of great importance.

### GATA1 directly binds to the core promoter of hCREG and regulates its activation

**Fig 4:**
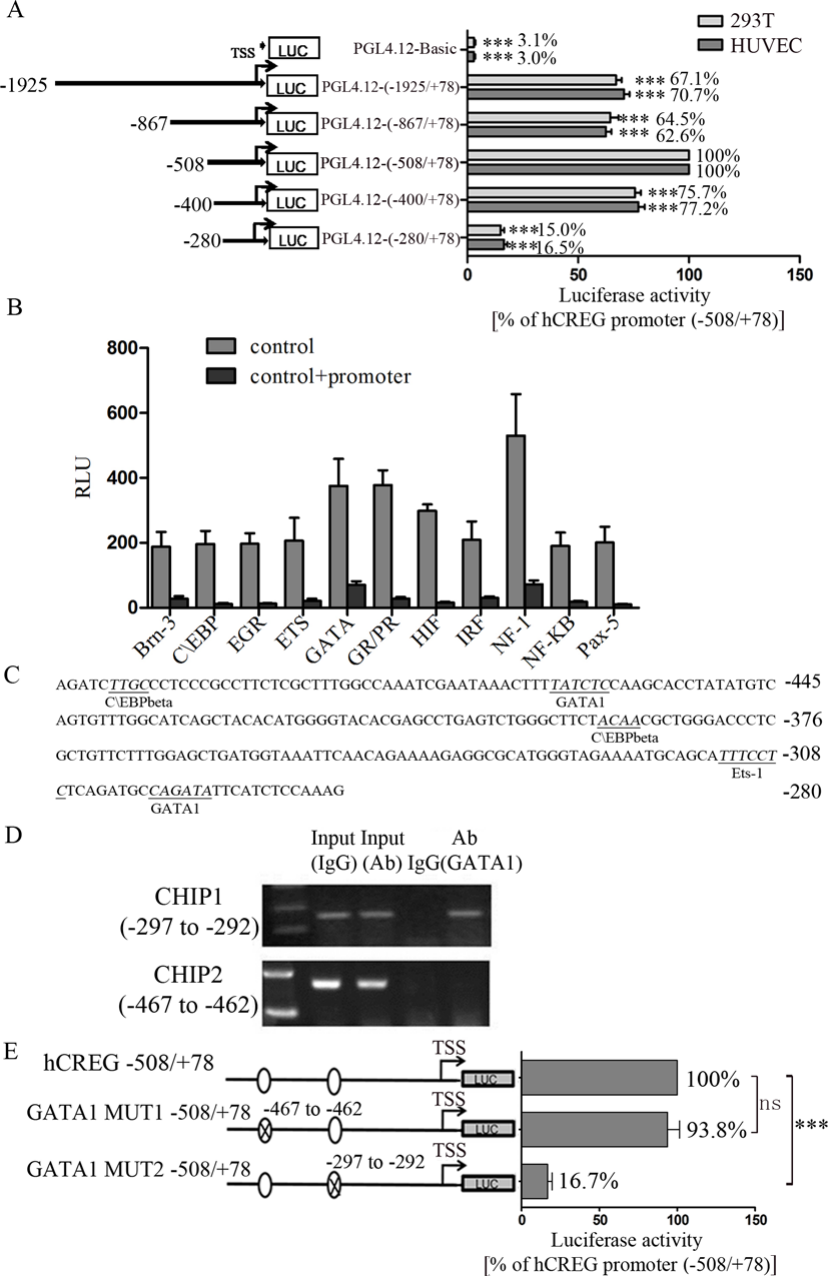
Identification of the core promoter of human CREG gene (hCREG) and the transcription factors that bind to the hCREG promoter. (A) Identification of the core promoter of hCREG. A 1.9 kb genomic DNA sequence upstream of the hCREG transcription start site (TSS) linked with Xho I/Hind III restriction sites was subcloned into pGL4.12-basic luciferase reporter vectors. A series of promoter deletions were created based on the 1.9 kb hCREG-luciferase reporter. HUVECs and 293T cells were transiently transfected with reporter vectors and harvested after 48 h. For each construct, a PGL4.73 plasmid was co-transfected to correct for differences in transfection efficiency. The corrected luciferase activity was normalized to the activity of the hCREG -508/+78 plasmids (100% activity). (B) Promoter-binding transcription-factor (TF) profiling array assay of hCREG core promoter was performed. This is a competitive binding assay performed to identify promoter-bound TFs through comparisons of the results in the presence (control + promoter) or absence (control) of the hCREG core promoter. If the hCREG promoter contains a TF binding sequence, it will display a lower chemiluminescence activity. In this study, hCREG was found to potentially bind with Brn-3, C\EBP, EGR, ETS, GATA, GR/PR, HIF, IRF, NF-1, NF-κB and Pax-5. (C) Bioinformatic predictions of the consensus binding sequences of C\EBP β, GATA1, and Ets-1. (D) ChIP assay confirmed that GATA1 bound directly to hCREG at the consensus GATA binding sequence [(-297/-292 bp) upstream from TSS]. (E) Plasmids containing the hCREG promoter (-508/+78) with 2 deletion mutations at the consensus GATA1 binding element were constructed with the luciferase (Luc) gene in PGL4.12 vectors. Luciferase activity was normalized to that of the hCREG core promoter (100%). n = 9. Data are shown as the mean ± SE from 3 independent experiments performed in triplicates. *P<0.05 and ***P<0.001 compared with either the hCREG core promoter (100%) (A and E) or the control (B).

To investigate the upstream factors regulating hCREG, we first generated luciferase constructs to determine the core promoter of the hCREG gene. As shown in Fig 4A, the hCREG 586 bp (-508/+78) fragment exhibited the highest promoter activity, which was defined as 100%, and this segment was identified to be the core promoter region. In addition, the 2003 bp (-1925/+78) fragment, 945 bp (-867/+78) fragment and 478 bp (-400/+78) fragment produced similar results and displayed relatively high promoter activities (70.7%, 62.6% and 77.2% in HUVECs and 67.1%, 64.5% and 75.7% in 293T cells, respectively), while the 358 bp (-280/+78) fragment had extremely low activity (16.5% in HUVECs and 15% in 293T cells), indicating that the fragment at -400/-281 may play a pivotal role in regulating hCREG transcription.

To characterize the transcription factors (TFs) that bind to the hCREG core promoter region (-508/+78) and regulate the activation of the hCREG gene, 48 TFs in HUVECs were assessed using a competitive promoter-binding TF profiling array. The results demonstrate the possible presence of binding sites for Brn-3, C\EBP, EGR, ETS, GATA, GR/PR, HIF, IRF, NF-1, NF-κB and Pax-5 within the hCREG core promoter (Fig 4B). Bioinformatic predictions showed there were 2, 1 and 2 consensus binding sequences for C\EBP β, GATA1, and Ets-1, respectively (Fig 4C). ChIP assay results further demonstrate that GATA1 bound to the hCREG promoter directly at the consensus binding sequence (-297/-292) rather than at -467/-462 (Fig 4D). To confirm this finding, reporter vectors of a mutant hCREG promoter with deletions of the 2 consensus GATA1 binding sequences [-467/-462 (GATA1-MUT1) and -297/-292 (GATA1-MUT2)] were constructed and transiently transfected into HUVECs to assess luciferase activity. Compared with the activity of the core promoter of hCREG (-508/+78), which was defined as 100%, the transcriptional activity of GATA1-MUT1 was almost no change (93.8%), while that of GATA1-MUT2 was dramatically reduced (83.3%) (Fig 4E). These data suggest that GATA1 can directly bind to a cis-acting DNA element in the hCREG promoter region, which is essential for the activation of hCREG transcription.

### Overexpression of GATA1 activates hCREG expression and inhibits HUVEC apoptosis induced by high glucose/high palmitate stimulation

**Fig 5:**
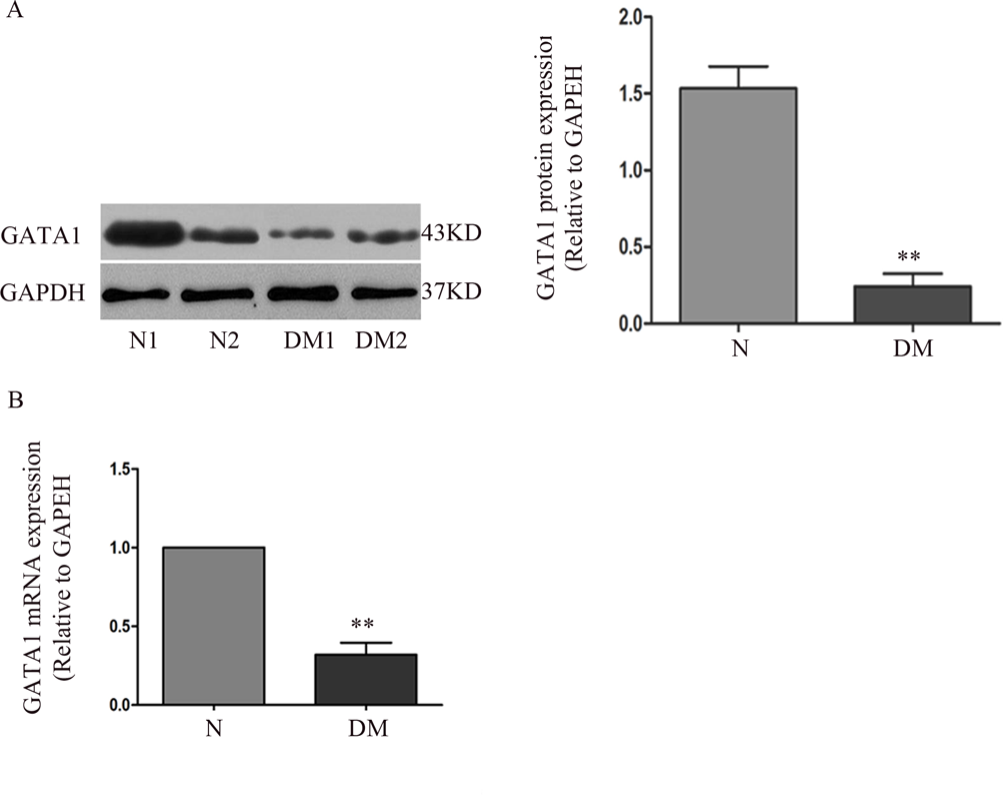
Expression of GATA1 in arteries from diabetic patients. (A) Expression of GATA1 in arteries from diabetic patients (DM1 and DM2) and in normal arteries (N1 and N2) assessed via immunoblotting. (B) Expression of GATA1 in arteries from diabetic patients assessed via real-time PCR. n = 4. GAPDH served as the control. Data are shown as the mean ± SE. **P<0.01 compared with control arteries (N).

To explicitly determine the role of GATA1 in relation to diabetic complications, human diabetic arteries were obtained and analysed. The expression of GATA1 was shown to be markedly decreased (84% and 78%) in diabetic arteries relative to the expression in arteries from non-diabetic subjects as assessed using immunoblotting (Fig 5A) or real-time PCR (Fig 5B), respectively, which indicates that GATA1 might be involved in the development of atherosclerosis in individuals with diabetes.

**Fig 6:**
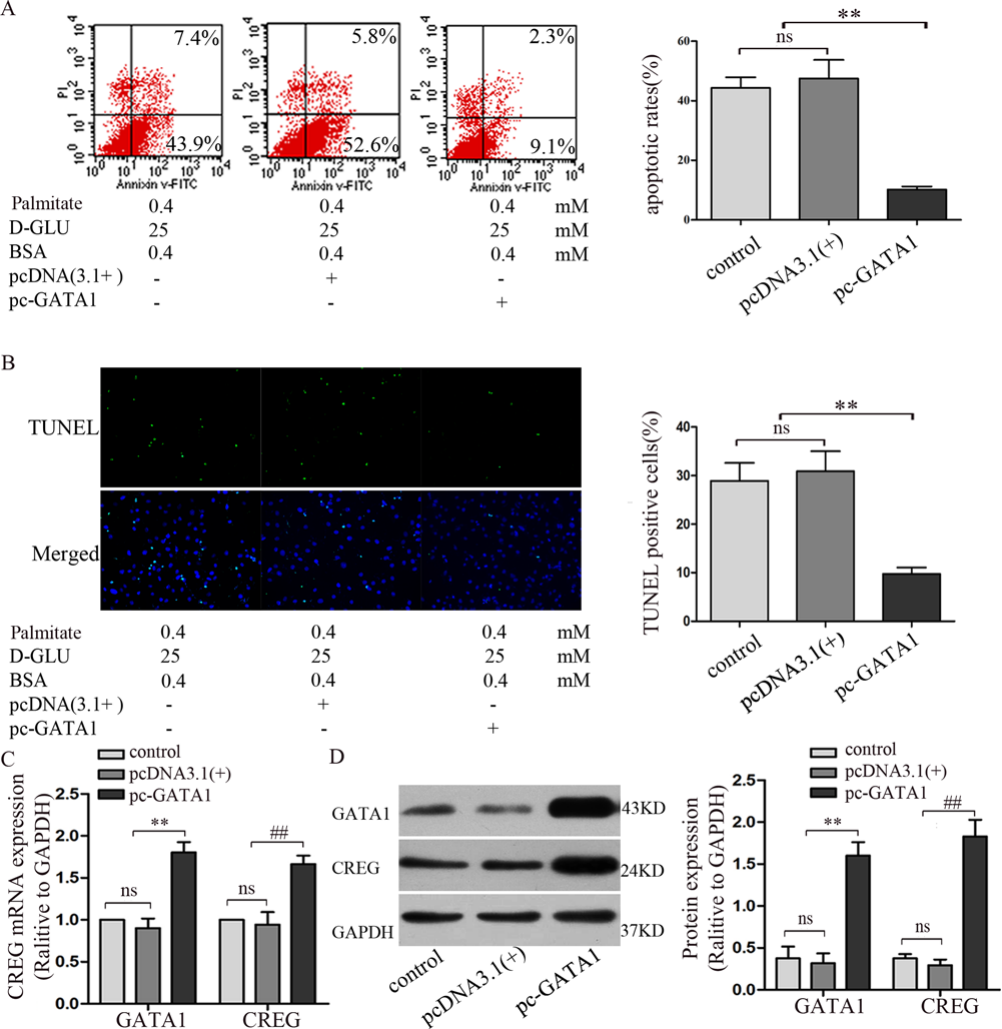
Effect of GATA1 over-expression on high glucose/high palmitate-induced apoptosis and CREG expression in HUVECs. GATA1 over-expression was achieved by transfecting HUVECs with pcDNA 3.1(+) containing full length human GATA1 cDNA. HUVECs or HUVECs transfected with empty pcDNA 3.1(+) were used as controls. Cells were treated with high glucose/high palmitate concentrations for 24 hours prior to the following analyses. (A) Apoptosis was assessed via Annexin V/PI dual-colour flow cytometry. Cells staining positive for Annexin V-FITC and negative for PI were considered to be undergoing apoptosis. (B) Apoptosis was assessed via TUNEL staining. (C) Expression of CREG and cleaved caspase-3 were determined via immunoblotting. (D) Expression of CREG and cleaved caspase-3 were determined via Real-time PCR. GAPDH was used as a loading control. n = 4. **, ##P<0.01 compared with control HUVECs or HUVECs transfected with the pcDNA 3.1 (+) vehicle control.

To determine the roles of GATA1 in hCREG expression and endothelial apoptosis, full-length human GATA1 cDNA (1245 bp) was amplified via PCR from whole HUVEC cDNA and inserted into the pcDNA3.1 (+) expression vector (Invitrogen) via EcoRI/XhoI double digestion. The plasmid pcDNA3.1 (+)-GATA1 was transfected into HUVECs and selected using 500 μg/ml G418, and cells transfected with an empty pcDNA3.1 (+) plasmid were used as a vehicle control. Then, cells were incubated with 25 mM D-glucose and 0.4 mM palmitate for 24 h to induce apoptosis. Annexin V/PI flow cytometry showed that the percentage of apoptotic cells was reduced by 47% in GATA1 over-expressing HUVECs (pc-GATA1) relative to the expression in the vehicle control [pcDNA3.1(+)] (Fig 6A). TUNEL staining also showed consistent results, indicating that apoptosis was decreased by 21% in GATA1 over-expressing HUVECs relative to expression in the control group (Fig 6B).

We further detected the expression of GATA1 and CREG at both the transcription and protein level. Real-time PCR analysis showed that GATA1 and CREG expression increased by approximately 2-fold in GATA1 over-expressing HUVECs relative to expression in the control group (Fig 6C). Immunoblotting showed that GATA1 and CREG expression increased approximately 4-fold relative to expression in the control group (Fig 6D).

These data demonstrate that the over-expression of GATA1 activates CREG expression, leading to the protection of HUVECs against high glucose/high palmitate-induced apoptosis.

## Discussion

Currently, accumulating evidence suggests that atherogenesis is stimulated by high circulating concentrations of glucose and FFAs, specifically palmitate. In the present study, we demonstrate for the first time that hCREG is transcriptionally activated by GATA1 and that regulation by this transcription factor helps protect the vascular endothelium of diabetic subjects against apoptosis. In this study, we obtained several novel findings that strongly support the presence of a protective role of CREG and its upstream regulation. Firstly, the expression of CREG was obviously decreased and endothelial apoptosis was increased in the atherosclerotic vasculature of diabetic patients when compared with the findings in non-diabetic control vessels. Secondly, under high concentrations of glucose (25 mM D-glucose) and different concentrations of palmitate (0.2, 0.3 or 0.4 mM), which simulate the pathological stimuli of poorly controlled T2DM in vivo, apoptosis increased and CREG expression decreased in HUVECs in a concentration dependent manner in vitro. Thirdly, elevating the expression of CREG antagonized HUVEC apoptosis, whereas suppressing CREG expression further increased HUVEC apoptosis induced by high glucose/high palmitate concentrations. Finally, GATA1 directly binds to the hCREG promoter and activates CREG expression, leading to the protection of HUVECs from the apoptosis induced by high glucose/high palmitate stimulation.

ECs play a major role in regulating blood vessel development, homeostasis and remodelling, and in cardiovascular pathologies by virtue of their location between the blood and the surrounding tissues[25, 26]. An increase in EC apoptosis is considered to be an early event in atherosclerosis and an initiator of its progression[27].Therefore, understanding the regulation of EC apoptosis, with the goal of intervening in this process, has become a current research focus.

Previous studies have reported that CREG might attenuate atherosclerotic endothelial apoptosis via the VEGF/PI3K/Akt pathway[15], but the upstream regulatory mechanism of the hCREG gene is unknown. In this study, we defined the core promoter elements (-508/78) of hCREG, including two activator fragments (-508/-401) and (-400/-281), and identified a consensus DNA binding site for GATA1 centred at -297 bp to -292 bp from the TSS. Both a promoter-binding TF profiling array assay and a ChIP assay demonstrated that GATA1 interacts specifically with the GATA1 binding site. The GATA family of transcription factors are important in mediating cell-specific gene expression. GATA1 is an important TF involved in the migration and apoptosis of ECs, which may be mediated by the regulation of the expression of AGGF1[28]. Additional experiments showed that removing the cis-acting DNA elements of GATA1 (-297 bp to -292 bp from the TSS) effectively reduced the expression of hCREG and resulted in increased cell apoptosis. These results suggest that GATA1 is an important regulator of the hCREG gene in its role in inhibiting endothelial apoptosis.

The limitations of our study are as follows. First, in addition to GATA1, several other transcription factors may positively and negatively regulate the expression of hCREG. As shown in our results, there is a trans-acting DNA element that represses the expression of hCREG located from -867 bp to -509 bp from the TSS. Future studies are needed to precisely define the minimum sequence length for this repressor and to identify potential TFs that bind to the site. In addition, the protective role and the underlying mechanisms of CREG demonstrated in our study are mainly from in vitro data, and these findings need to be further validated in in vivo settings using suitable T2DM animal models that have been genetically modified in relation to their CREG or GATA1 expression.

## Conclusions

In conclusion, a reduction in CREG expression causes endothelial apoptosis that is induced by diabetes-related stimuli in vitro, which can be rescued by the over-expression of CREG. The upregulation of CREG can be achieved by the activation of GATA1, which binds directly to the promoter of CREG. CREG might be a therapeutic target for the management of macrovascular complications in diabetic patients.
